# The Kiss Switch Brings Inactive R3C Ligase Ribozyme Back to Life

**DOI:** 10.3390/biology7010007

**Published:** 2018-01-09

**Authors:** Kana Tanizawa, Sayuri Uchida, Eri Kurihara, Takuya Umehara, Koji Tamura

**Affiliations:** 1 Department of Biological Science and Technology, Tokyo University of Science, 6-3-1 Niijuku, Katsushika-ku, Tokyo 125-8585, Japan; kanakana5629.pooh@icloud.com (K.T.); uchidasayuri@gmail.com (S.U.); kurieri123@yahoo.co.jp (E.K.); tumehara@rs.noda.tus.ac.jp (T.U.); 2 Research Institute for Science and Technology, Tokyo University of Science, 2641 Yamazaki, Noda, Chiba 278-8510, Japan

**Keywords:** R3C ligase ribozyme, kissing-loop interaction, activity, RNA switch, RNA world

## Abstract

R3C ligase ribozyme catalyzes the nucleophilic attack by a 3′-hydroxyl on a 5′-α-phosphorus of triphosphates to form a 3′-5′-phosphodiester bond. In the present study, although the truncation of R3C ribozyme was accompanied by a large reduction in ligation activity (decrease by two orders of magnitude compared to that of the ligated product of full-length R3C ribozyme after 18.5 h at 23 °C), the introduction of complementary seven-membered kissing-loops served as a “switch” to reactivate the truncated R3C ribozyme with approximately one-fifth of the activity of the full-length R3C ribozyme. This reactivation occurred in a trans-manner, and the grip region and substrate-binding site of the truncated R3C ribozyme were necessary to locate the substrate in the proper position for ligation with the other molecule. Reactivation resulted from complex tertiary interactions between two ribozymes, including kissing-loop interaction-induced annealing and the formation of a stable duplex. The drastic increase of the activity of poorly active ribozymes through the kissing-loop interaction may provide an important clue into the acquisition of substantial activity during the evolution of the RNA world.

## 1. Introduction

The discovery of ribozymes [1,2] was an epoch-making event, resulting in a shift in the general concept of the understanding of life itself; RNA plays a dual role in information storage and catalysis, which has led to the “RNA world” hypothesis [3]. Several natural and artificial ribozymes catalyzing many steps of biological reactions have been identified to date. The ribozymes responsible for protein synthesis are particularly important because they might represent crucial molecules that contributed to the transition from an RNA world to an RNA/protein world [4,5,6]. However, before this transition, the nucleotides would have to be polymerized to certain threshold lengths so as to establish a functional RNA world. Joyce and Orgel suggest that a triple stem loop containing 40 to 60 nucleotides is the minimum required length for RNA catalytic activity [7].

**Figure 1:**
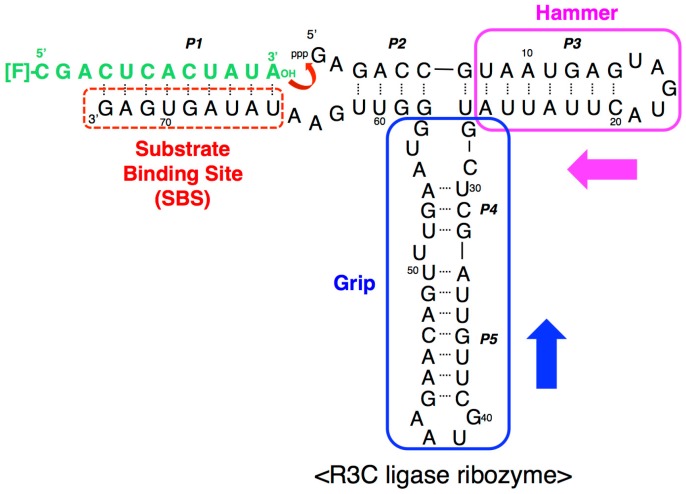
Composition of the R3C ligase ribozyme with a fluorescence-labeled RNA substrate. The ribozyme is composed of 73 nucleotides and forms five paired regions (P1–P5). The stem-loop region comprising P3 was designated as the “hammer”, and the region comprising P4 and P5 was designated as the “grip” (see above). R3C ribozyme has a single-stranded substrate-binding site (SBS) at the 3′-terminal region. In a previous study [12], we created a smaller R3C variant (~50 nucleotides) which still retained greater than 10% activity of full-length R3C by shortening both the “hammer” and “grip” [12]. The numbering of the nucleotides starts from the guanosine triphosphate at the 5′-end.

**Figure 2:**
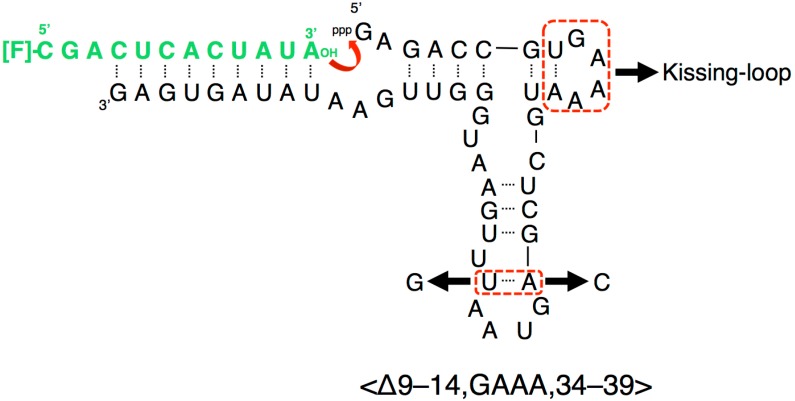
Composition of a variant of the R3C ligase ribozyme, <∆9–14,GAAA,34–39>, with a fluorescence-labeled RNA substrate [12]. Although the addition of only one more base pair to the “grip” region showed greater than 10% activity of the full-length R3C (<∆9–14,GAAA,35–39>), <∆9–14,GAAA,34–39> showed a drastic decrease of activity [12]. We introduced seven-membered single-stranded nucleotides, expecting the inter-molecular interaction through the kissing-loop, and replaced the A–U pair at the end of the grip stem with C–G. Because U51 is proximal to U50 of the A33–U50 pair, the A33–U51 pair can also be stacked on P4; however, this is not possible with the C–G pair (nucleotide position from Figure 1).

Several RNA ligase ribozymes have been produced using in vitro selection techniques [8], such as class I ligase ribozyme [9], L1 ligase ribozyme [10], and R3C ligase ribozyme [11]. The R3C ribozyme is the smallest among them and was originally composed of 73 nucleotides [11], in which a three-way junction structure is formed around three stems (P2, P3, P4) (Figure 1). By partially deleting both the “grip” (P4 + P5) and “hammer” (P3) stem-loops, we generated a truncated R3C variant (<∆9–14,GAAA,35–39>, 50 nucleotides) which still retained greater than 10% activity of a full-length R3C ribozyme [12]. However, a single base-pair deletion from the “grip” region (<∆9–14,GAAA,34–39>, 48 nucleotides) drastically decreased its activity to two orders of magnitude less than that of the ligated product of the full-length R3C ribozyme after 18.5 h at 23 °C (Figure 2) [12]. The acquisition of sufficient activity in ribozymes is considered to be a crucial step in the evolution of the RNA world. Here, we demonstrate that the inter-molecular interaction through the kissing-loop contributes to this dramatic increase in the ligation activity of this shortened R3C ribozyme with poor activity (Figure 2).

## 2. Materials and Methods

### 2.1. Plasmid Construction and In Vitro Transcription

Unlabeled deoxyribonucleotides were synthesized by Eurofins Genomics K.K. (Tokyo, Japan). High-performance liquid chromatography-purified 5′-terminal 6-carboxyfluorescein (6-FAM)-labeled oligonucleotides were prepared by Japan Bio Services Co., Ltd. (Saitama, Japan). Each template DNA was prepared from chemically synthesized deoxyribonucleotides carrying the T7 promoter and the sequences corresponding to variants of the R3C ligase ribozyme, along with two synthetic primers for polymerase chain reaction amplification. RNA transcription was performed at 37 °C for 16 h or at 42 °C for 3 h in a reaction mixture containing 40 mM Tris-HCl (pH 8.0), 10 mM dithiothreitol, 2 mM spermidine, 8 mM MgCl_2_, 2.5 mM each nucleoside triphosphate (ATP, CTP, GTP, UTP), template DNA (0.2 mg/mL), and pure T7 RNA polymerase (~100 μg/mL) [13]. The transcripts were purified by denaturing 12% polyacrylamide gel electrophoresis. The concentrations of the obtained purified RNA were determined from the ultraviolet absorbance at a wavelength of 260 nm using an Implen NanoPhotometer (München, Germany).

### 2.2. Analysis of Ligation

Ligation analysis was performed according to the method of Rogers and Joyce with a slight modification [11,12]. The R3C ligase ribozyme or its variants, dissolved in solution containing 50 mM Tris-HCl (pH 8.5) and 15 mM MgCl_2_, were first heated to 37 °C for 5 min and then cooled to 4 °C. Then, the ligation reaction was started by adding 1.5 μL of 50 μM substrate to the solution. The final concentration of each ribozyme and the substrate was 5 μM each. After incubation at 23 °C, 15 μL of aliquots were removed at specific time points, put into the same volume of 7 M urea and 0.08% (*w*/*v*) bromophenol blue, and stored at –20 °C for quenching the reaction. The solution was then applied onto a denaturing 12% polyacrylamide gel for electrophoresis. The gel was analyzed on a Typhoon FLA 7000 system (GE Healthcare Japan, Tokyo, Japan), and the ligated products were quantified using Image Quant TL software. The measurements were obtained three times. Representative gel images were shown.

### 2.3. Electrophoretic Mobility Shift Assay (EMSA)

<A> or <U> (40 mM) or the mixture of 40 mM each of <A> and <U> in 50 mM Tris-HCl (pH 8.5) and 15 mM MgCl_2_ was first heated to 37 °C for 5 min. Then, the solution was incubated at 23 °C for 18.5 h. After the addition of an equal volume of 10% glycerol, the solution was analyzed by electrophoresis on non-denaturing 8% polyacrylamide gels in 50 mM Tris-HCl (pH 8.5) and 15 mM MgCl_2_ [13,14]. The gel was stained with 0.04% toluidine blue.

## 3. Results

**Figure 3:**
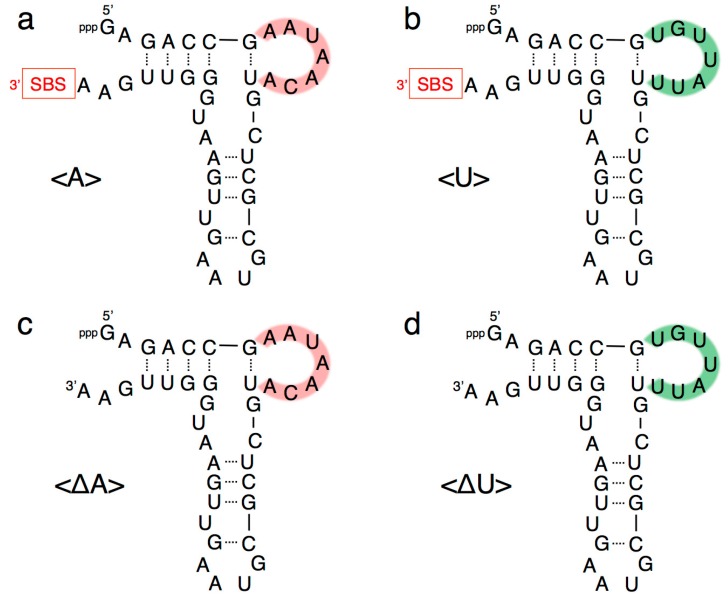
Composition of variants of R3C ligase ribozyme (**a**) <A>, (**b**) <U>, (**c**) <∆A>, and (**d**) <∆U> used in this study. Complementary seven-membered single stranded nucleotides (5′-AAUAACA-3′ and 5′-UGUUAUU-3′) are colored in pink and green, respectively. SBS, 3′-terminal substrate-binding site.

Our previous study indicated that a grip- and hammer-partially truncated mutant (<∆9–14,GAAA,34–39>) retained slight ligation activity (Figure 2) [12]. Based on this less-active variant, in the present study we first prepared two new mutants, designated <A> and <U>, which possessed complementary seven-membered single-stranded nucleotides (5′-AAUAACA-3′ and 5′-UGUUAUU-3′) that replaced the original GAAA tetraloop and a U–A pair (Figure 2 and Figure 3a,b). In addition, the A–U pair at the end of the grip-stem of the <∆9–14,GAAA,34–39> (corresponding to the original position 33) [12] was replaced with C–G (Figure 2 and Figure 3a,b) to stabilize the structure around the region.

**Figure 4:**
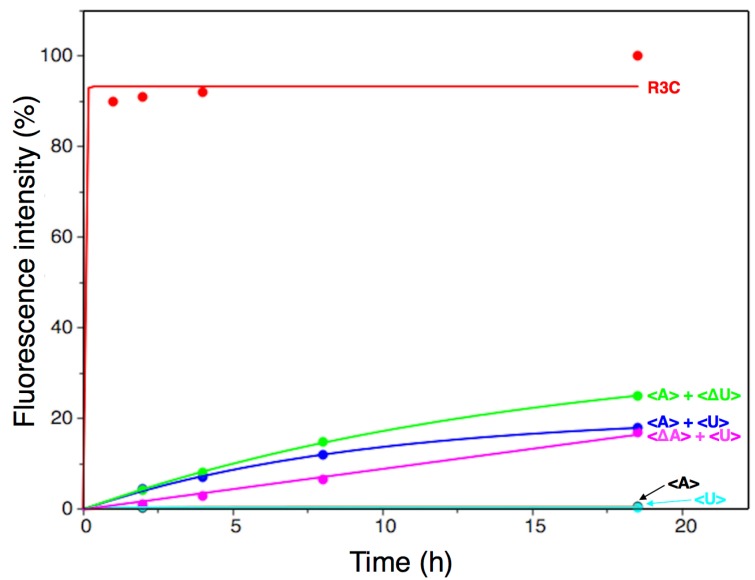
Analysis of the ligation product using a fluorescence-labeled RNA substrate (5′-CGACUCACUAUA-3′) with a full-length R3C ligase ribozyme, or the combinations of <A>, <U>, <∆A>, and <∆U>. After incubation at 23 °C for the indicated times, 15-μL aliquots were removed, put into the same volume of 7 M urea and 0.08% (*w*/*v*) bromophenol blue, and stored at –20 °C in order to quench the reaction. The solution was then applied to denaturing 12% polyacrylamide gel electrophoresis. The gel was visualized on Typhoon FLA 7000 (GE Healthcare Japan, Tokyo, Japan) and the ligated products were quantified using Image Quant TL software and plotted on the graph. The data were fit to a single exponential curve.

The ligation activities of <A> or <U> alone were drastically decreased by two orders of magnitude, compared to that of the full-length R3C ligase ribozyme, as evident from the data corresponding to the last time point (Figure 4, 18.5 h). This finding is concurrent with a previously reported conclusion that <∆9–14,GAAA,34–39> showed low activity [12]. This means that the replacement of the GAAA tetraloop and a U–A pair with seven-membered single-stranded nucleotides did not apparently increase the ligation activities, i.e., slight activities were detected (Figure 4). However, surprisingly, as shown by the time course analysis of the ligation (Figure 4), the mixture of <A> and <U> drastically increased the ligation activity.

**Figure 5:**
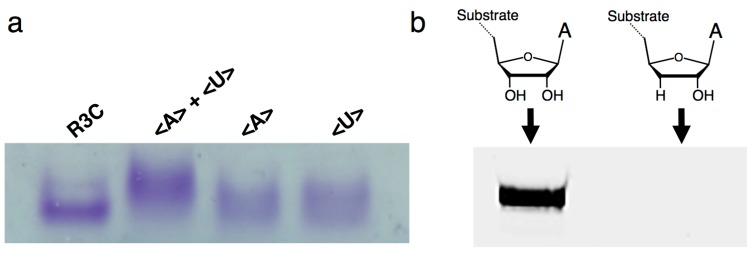
(**a**) Electrophoretic mobility shift assay of the (from left to right) full-length R3C ligase ribozyme, mixture of <A> and <U>, <A> alone, and <U> alone. The nucleotides were separated via native 8% polyacrylamide gel electrophoresis. The gel was stained with 0.04% toluidine blue. (**b**) Analysis of the ligation product by the R3C ribozyme using an RNA substrate possessing 3′-deoxyadenosine at the 3′-terminal (5′-CGACUCACUAUA(3′-deoxy)-3′). The volume of the reaction mixture was 15 μL and the incubation was performed at 23 °C for 18.5 h. The gel was visualized on Typhoon FLA 7000 (GE Healthcare Japan, Tokyo, Japan).

Native gel electrophoresis indicated that the mixtures of <A> and <U> behaved as dimers by the introduction of complementary seven-membered kissing-loops (Figure 5a). In addition, when we used the RNA substrate possessing a 3′-deoxy adenosine at the 3′-terminal, the ligation activity was greatly decreased (Figure 5b), suggesting that the ligation site is 3′-OH and that the final product possesses 3′–5′ linkage, as in the original R3C ribozyme [11,12].

**Figure 6:**
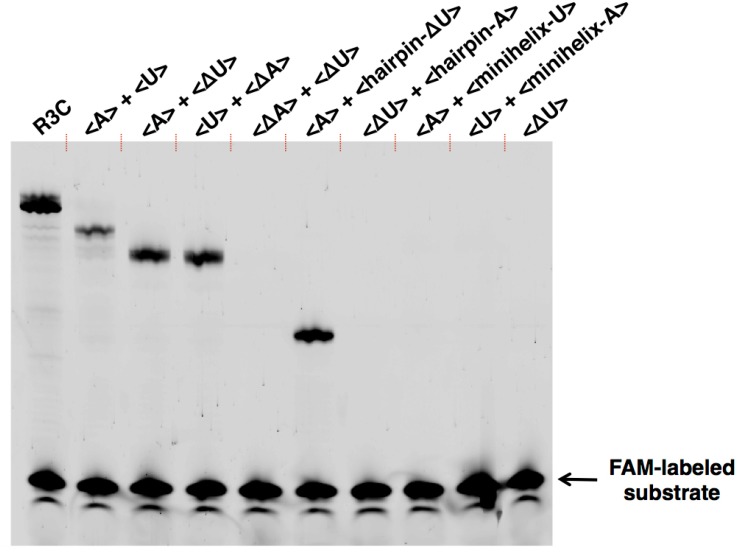
Ligation activities of the combinations of <A>, <U>, <∆A>, <∆U>, <hairpin-A>, <hairpin-∆U>, <minihelix-A>, and <minihelix-U>. The sequence of each RNA is described in Figure 1, Figure 3, and Figure 7. The volume of the reaction mixture was 15 μL and the incubation was performed at 23 °C for 18.5 h. The solution was put into the same volume of 7 M urea and 0.08% (*w*/*v*) bromophenol blue and then applied to denaturing 12% polyacrylamide gel electrophoresis. The gel was visualized on Typhoon FLA 7000 (GE Healthcare Japan, Tokyo, Japan).

As in the full-length R3C ligase ribozyme (composed of 73 nucleotides), <A> and <U> (Figure 3a,b) each possess a 3′ single-stranded structure at the site of substrate hybridization, i.e., the substrate-binding site (SBS) (Figure 1, P1 region). After deleting the SBS of <A> or <U> to generate two new variants designated <∆A> and <∆U>, respectively (Figure 3c,d), the mixture of <A> and <∆U>, or that of <∆A> and <U>, also showed similar activity to that observed with the mixture of <A> and <U>, which was in contrast with the case of <A> or <U> alone (Figure 4). In addition, the ligation occurred on the SBS-null variants (<∆A> and <∆U>), as evident from the size of the bands on the denaturing polyacrylamide gel electrophoresis (Figure 6).

**Figure 7:**
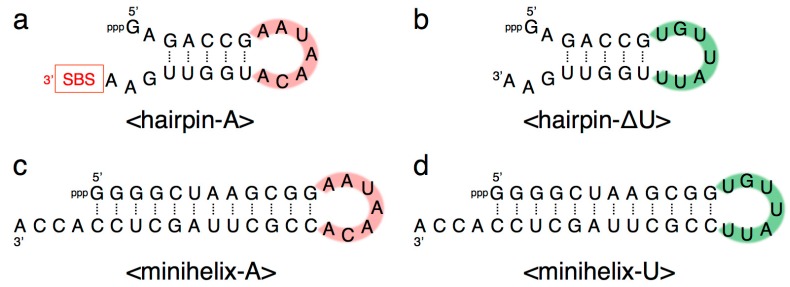
Composition of (**a**) <hairpin-A>, (**b**) <hairpin-∆U>, (**c**) <minihelix-A>, and (**d**) <minihelix-U>. Complementary seven-membered single stranded nucleotides (5′-AAUAACA-3′ and 5′-UGUUAUU-3′) are colored in pink and green, respectively. SBS, 3′-terminal substrate-binding site.

We further generated two variants of R3C ribozyme with no “grip” (<hairpin-A> and <hairpin-∆U>) (Figure 7a,b). <hairpin-A> contained the SBS; however, <hairpin-∆U> did not. Unexpectedly, the mixture of <A> and <hairpin-∆U> caused 6-carboxyfluorescein (6-FAM)-labeled substrate ligation on <hairpin-∆U> despite the absence of SBS in this truncated molecule. However, the mixture of <∆U> and <hairpin-A> did not yield any large ligation products (Figure 6).

We next designed <minihelix-A> and <minihelix-U>, which corresponded to a minihelix^Ala^ (a coaxial stack of the acceptor stem on the T-stem of *E. coli* tRNA^Ala^) [15,16,17,18]; however, the loop region were replaced with 5′-AAUAACA-3′ and 5′-UGUUAUU-3′, respectively (Figure 7c,d). The mixture of <A> and <minihelix-U> caused a drastic decrease of the ligation activity compared with that of the mixture of <A> and <∆U>, or the mixture of <U> and <∆A>, and the level was comparable to that detected in the case of <A> alone (Figure 6). Similar low activity was found in the case of the mixture of <U> and <minihelix-A> (Figure 6).

## 4. Discussion

The results of this study indicate that inter-molecular interactions caused by the introduction of the kissing-loop drastically increased the ligation activity of a truncated R3C ribozyme that showed poor activity previously (Figure 4 and Figure 6). Our newly designed complementary seven-membered loops of <A> and <U> successfully formed dimers (Figure 5a). The present results indicate that even if a truncated ribozyme retains slight activity, the activity can be drastically increased through simple mutual interactions.

**Figure 8:**
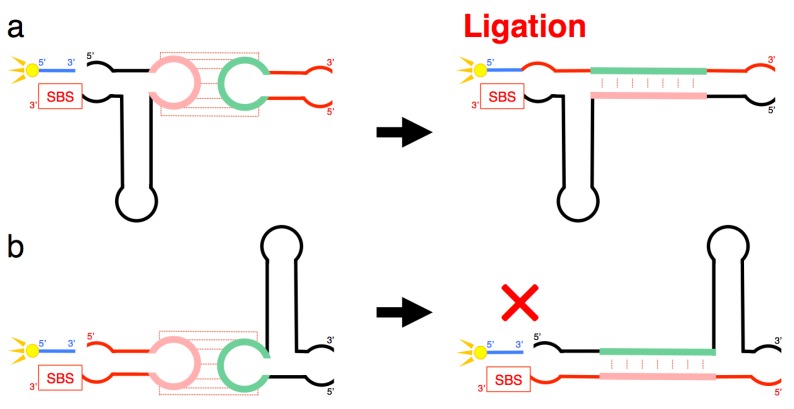
(**a**) Schematic representation of a process that may occur when <A> and <hairpin-∆U> combine. Although <A> contains the SBS, substrate ligation occurred on <hairpin-∆U> despite the absence of SBS in this molecule (Figure 6). (**b**) Schematic representation of a process that may occur when <∆U> and <hairpin-A> combine. Any large substrate ligation products were not detected in this combination, although <hairpin-A> contained the SBS (Figure 6). Both (**a**,**b**) suggest complex tertiary interactions between the two ribozymes, including kissing-loop interaction-induced annealing and the formation of a stable duplex, which partially rescued the activity of the truncated ribozyme.

Importantly, the improvement of activity occurred in a trans-manner (Figure 6 and Figure 8). The site of ligation was located at the 5′-end of the other ribozyme that was different from the substrate-bound ribozyme. This is evident from the combination of <A> and <hairpin-∆U> yielding the ligation product (Figure 6). However, the combination <∆U> and <hairpin-A> did not yield any large ligation products (Figure 6). These results suggest that the grip region and substrate-binding site of the truncated R3C ribozyme are necessary to locate the substrate in the proper position for ligation with the other molecule. Furthermore, the 5′-end and its vicinity are also important for the conformation of the site of ligation composed of both ribozymes. The success of <hairpin-∆U> and the failure of <minihelix-U> to rescue the activity of <A> suggested complex tertiary interactions between the two ribozymes, including kissing-loop interaction-induced annealing and the formation of a stable duplex (Figure 8). Human immunodeficiency virus type 1 (HIV-1) is known to form two types of dimers through kissing-loop-mediated interactions. In this case, interactions between the two loops initially generate unstable dimers; the formation of these dimers then induces annealing between the two stems to form stable intra-strand dimers [14,19,20,21,22]. A similar rearrangement may have occurred in our experimental system.

The chemistry of the ligation catalyzed by R3C ribozyme is considered to be identical to that catalyzed by natural RNA and DNA polymerases [23,24]: a nucleophilic attack by a 3′-hydroxyl of the substrate on a 5′-α-phosphorus of guanosine triphosphates of the ribozyme to form a 3′-5′-phosphodiester bond with the concomitant release of pyrophosphate. The proteinaceous enzymes use a two-Mg^2+^-ion mechanism: one Mg^2+^ lowers the p*K*_a_ of the 3′-hydroxyl for the in-line nucleophilic attack, while the other Mg^2+^ assists in the pyrophos­phate release by stabilizing the negative charge [25]. Kissing-loop interaction typically involves complete base pairing and continuous stacking of helices [26,27]. The base pairing through the loop regions of <A> and <U>, with the effect of stacking, may make the deleted “hammer” region more “rigid”, which could trigger the rearrangement of the complex and ensure the proper positioning of the reactants and Mg^2+^ ions.

The length of the nucleotides is a key factor for establishing the RNA world. We previously shortened the R3C ribozyme up to ~50 nucleotides, which is the size formed by the catalytic functions of montmorillonite [28]. However, in general, primitive ribozymes, if they were formed, would have shown limited and weak activity; thus, the ability of such small-sized RNA to acquire improved activity would have been a crucial point in the formation of the primitive biological system based on the RNA world. Kissing-loop interaction is one of the typical RNA–RNA interactions. In addition to the example of HIV-1 described above [14,19,20,21,22], this interaction also occurs in the complex formed between ColE1 plasmid RNAs I and II [29]. Furthermore, a kissing-loop interaction between the substrate and the catalytic domain of the Varkud satellite ribozyme contributes to the rearrangement of the substrate helix into a conformation that is critical for substrate binding and activation [30]. In the substrate stem loop I (SLI)-stem loop V (SLV) kissing-loop junction of the ribozyme, SLV has been shown to act as a scaffold to provide stability to the junction and the Mg^2+^ ions associated with SLV [31]. Thus, the kissing-loop interaction may provide important clues into evolution of the RNA world. Structural analysis in the future will help to elucidate the details of the mechanism.

## 5. Conclusions

Introduction of complementary seven-membered kissing-loops served as a “switch” to reactivate the truncated R3C ligase ribozyme with poor activity. This reactivation resulted from complex tertiary interactions between two ribozymes, including kissing-loop interaction-induced annealing and the formation of a stable duplex. The grip region and substrate-binding site of the truncated R3C ribozyme were necessary to locate the substrate in the proper position for ligation with the other molecule. Thus, the activity can be drastically increased through simple mutual interactions between small-sized RNAs and the kissing-loop interaction may provide a new perspective on the evolution of the RNA world.
